# Opioid-free analgesia with esketamine-dexmedetomidine versus opioid-based analgesia after gynaecological laparoscopic surgery: a double-blind, randomized, controlled trial

**DOI:** 10.1080/07853890.2026.2732292

**Published:** 2026-09-16

**Authors:** Ying Ren, Yu-Ting Li, Qiao-Li Yin, Hong-Ping Li, Peng-Ju Zhang, Jia-Hao Cao, Bin Yu, Xiao-Long Yang, Zi-Zhong Yang, Hao Kong

**Affiliations:** a Department of Anesthesiology, Peking University First Hospital, Beijing, China; b Department of Anesthesiology, Peking University First Hospital Ningxia Women and Children’s Hospital, Yinchuan, China

**Keywords:** Dexmedetomidine, esketamine, postoperative analgesia, postoperative quality of recovery, gynaecological laparoscopic surgery

## Abstract

**Background:**

Postoperative opioid-based analgesia after gynecological laparoscopic surgery often causes dizziness, nausea and vomiting, with limited benefit for mood and sleep. We tested whether opioid-free analgesia combining dexmedetomidine and esketamine was non-inferior to sufentanil-based opioid analgesia for pain control and superior for postoperative recovery quality.

**Methods:**

Adult ASA I–III patients undergoing elective gynecological laparoscopic surgery were randomized 1:1. The opioid-free group received patient-controlled intravenous analgesia (PCIA) with esketamine 1 mg kg^-1^ plus dexmedetomidine 3 μg kg^-1^; the opioid group received sufentanil 2 μg kg^-1^. The primary non-inferiority endpoint was the area under the curve (AUC) of numerical rating scale (NRS) pain scores during coughing within 48 h postoperatively; the primary superiority endpoint was the mean quality of recovery (QoR)-15 score on postoperative days 1 and 2.

**Results:**

Of 134 randomized patients, 132 were analyzed per intention-to-treat. Pain control was non-inferior in the opioid-free group (AUC 175 [47] vs 169 [37]; mean difference 6, 95%CI −8 to 20; non-inferiority margin 24, *p* = 0.008). The mean QoR-15 score was significantly higher in the opioid-free group (116 [10] vs 108 [12]; mean difference 8, 95%CI 4–12; *p* < 0.001), driven by better physical comfort and emotional state domain scores, with comparable pain domain scores. No serious adverse events occurred.

**Conclusions:**

Postoperative opioid-free analgesia with dexmedetomidine and esketamine provides analgesia non-inferior to sufentanil-based opioid analgesia and significantly improves postoperative quality of recovery, and may serve as a promising alternative strategy for postoperative analgesia in this population.

Trial registration: This trial was registered at the Chinese Clinical Trial Registry (identifier: ChiCTR2300078569) on 12 December 2023 before patient recruitment.

## Introduction

In the era of enhanced recovery after surgery (ERAS), the routine application of opioids in the perioperative period has been increasingly scrutinized, mainly due to a range of adverse effects including postoperative nausea and vomiting (PONV), hyperalgesia, respiratory depression, gastrointestinal hypomotility, immunosuppression, chronic pain and opioid dependence [1,2]. Opioid-free analgesia has emerged as a promising strategy in postoperative pain management. A growing body of evidence supports the safety and feasibility of opioid-free analgesic regimens across various surgical disciplines [3,4]. Common agents used in opioid-free protocols include dexmedetomidine, N-methyl-D-aspartate receptor antagonists (such as ketamine or esketamine), lidocaine, magnesium sulphate and regional anaesthesia techniques [5].

Dexmedetomidine is a highly selective α_2_-adrenoceptor agonist that provides sedative, analgesic and anxiolytic effects with minimal respiratory depression [6]. When used as an adjuvant in postoperative patient-controlled analgesia, dexmedetomidine reduces pain scores and opioid consumption [7]. It also offers additional benefits including reduced anxiety, lower PONV incidence, decreased shivering, relief of catheter-related discomfort, improved sleep quality and decreased risk of postoperative neurocognitive disorders [8].

Esketamine, a non-competitive N-methyl-D-aspartate receptor antagonist, exerts potent analgesic and antihyperalgesic effects at subanaesthetic doses and is recommended as a component of multimodal analgesia [9]. Building on the established antidepressant properties of ketamine [10], esketamine has been shown to prevent postoperative depression, negative mood disturbances and fatigue syndrome [11–13]. Perioperative administration of ketamine or esketamine also improves early subjective postoperative recovery and psychological well-being without increasing adverse events [14]. A recent clinical trial indicated that intraoperative infusion of esketamine reduces postoperative sleep disturbance in patients undergoing gynaecological laparoscopy [15].

Patients undergoing gynaecological laparoscopic surgery face unique postoperative challenges, including moderate acute pain, high susceptibility to PONV, increased anxiety and depressive symptoms and frequent sleep disruption [16]. Conventional opioid-based analgesia provides effective pain relief but may aggravate dizziness and PONV, with little beneficial effect on mood or sleep. Combined use of dexmedetomidine and esketamine addresses these limitations through synergistic pharmacologic actions: their analgesic, antiemetic, mood-regulating and sleep-improving effects are enhanced, while adverse reactions are mutually attenuated [17]. However, direct evidence supporting this combination as a postoperative opioid-free analgesic regimen remains limited. As intraoperative sufentanil and remifentanil remain standard components of balanced general anaesthesia, this trial specifically evaluates a postoperative opioid-free strategy rather than opioid-free perioperative management. The present double-blind, randomized controlled trial tested two hypotheses: (i) postoperative opioid-free analgesia using esketamine plus dexmedetomidine is non-inferior to opioid-based analgesia in analgesic efficacy; (ii) this postoperative opioid-free strategy significantly improves postoperative quality of recovery.

## Methods

### Study design and ethical approval

This was a single-centre, double-blind, randomized controlled trial conducted at a tertiary hospital. Ethical approval for this study (No.: KJ-LL-2023-60) was provided by the Ethical Committee of Peking University First Hospital Ningxia Women and Children’s Hospital, Yinchuan, China (Chairperson Prof Ying-Dong He) on 21 September 2023. The study was performed in accordance with the ethical principles of the Declaration of Helsinki. This trial was registered at the Chinese Clinical Trial Registry (identifier: ChiCTR2300078569) on 12 December 2023 before patient recruitment. Written informed consent was acquired from all participants. The study was performed in accordance with the CONSORT guidelines.

### Participants

We screened patients scheduled for elective gynaecological laparoscopic surgery in Peking University First Hospital Ningxia Women and Children’s Hospital. Inclusion criteria were adult patients with an ASA grade of I-III who received postoperative patient-controlled intravenous analgesia (PCIA). Exclusion criteria were: (i) pre-existing chronic pain; (ii) language, psychological or comprehension barriers; (iii) long-term analgesic use, opioid abuse, drug addiction or alcoholism; (iv) severe psychiatric disorders; (v) severe sinus bradycardia (heart rate < 50 beats·min^−1^), sick sinus syndrome, second-degree or higher atrioventricular block without pacemaker implantation, or preoperative left-ventricular ejection fraction < 30% and (vi) known allergy to study medications.

### Randomization and blinding

Randomization sequences were generated by a professional biostatistician using SAS version 9.2 (SAS Institute, Cary, NC, USA) with a 1:1 allocation ratio. Randomization lists were kept in sequentially numbered, sealed opaque envelopes by an independent research coordinator. During the study, envelopes were opened after induction of anaesthesia according to the recruitment order. The independent coordinator prepared study medications to ensure the identical appearance for postoperative PCIA and did not participate in anaesthesia, perioperative care or postoperative follow-up. Patients, anaesthetists, other clinical staff and investigators involved in recruitment, data collection and follow-up remained blinded to group assignment throughout the study.

### Anaesthesia, perioperative care and intervention

Standard intraoperative monitoring included electrocardiography, pulse oximetry, non-invasive blood pressure, capnography, bispectral index (BIS) and nasopharyngeal temperature. General anaesthesia was induced with midazolam (0.5 mg kg^−1^), propofol (1.0–2.0 mg kg^−1^), sufentanil (0.3 μg kg^−1^) and either rocuronium (0.6 mg kg^−1^) or cisatracurium (0.2 mg kg^−1^). Total intravenous anaesthesia was maintained by continuous infusion of propofol and remifentanil. Anaesthetic depth was adjusted to maintain BIS values between 40 and 60. Hemodynamic stability was maintained by keeping blood pressure within ± 30% of baseline values. Active warming was applied to maintain nasopharyngeal temperature above 36 °C. Fluid therapy and transfusion were administered according to routine clinical practice. For PONV prophylaxis, dexamethasone (5 mg) was routinely administered before induction, and a 5-hydroxytryptamine receptor antagonist was given intraoperatively. Non-steroidal anti-inflammatory drugs (NSAIDs) were administered routinely in the absence of contraindications.

At the end of surgery, patients were extubated and transferred to the postanaesthesia care unit (PACU). PCIA was initiated after extubation. In the opioid-free group, the analgesic solution contained esketamine 1 mg kg^−1^ and dexmedetomidine 3 μg kg^−1^, diluted to 100 ml with 0.9% sodium chloride. In the opioid group, the solution contained sufentanil 2 μg kg^−1^ only, diluted to 100 mL with 0.9% sodium chloride. The pump was set to deliver 2-mL boluses with an 8-min lockout interval and a background infusion of 1 ml h^−1^. Study PCIA was continued for 48 h postoperatively. Patients who exhausted study medication within 48 h or required additional analgesia after 48 h received the same formulation. Patients were monitored intermittently for blood pressure, heart rate and pulse oximetry until the next morning and twice daily on the ward until discharge.

### Measurements and outcomes

Preoperative pain intensity was assessed using the numerical rating scale (NRS; 0 = no pain, 10 = worst pain) at rest and during coughing. Preoperative sleep quality was assessed using the Pittsburgh Sleep Quality Index (PSQI). Preoperative anxiety and depression were evaluated using the Hospital Anxiety and Depression Scale (HADS). Baseline QoR-15 score was also measured preoperatively.

Postoperatively, NRS pain scores were assessed at PACU discharge, 12 h, 24 h, 36 h and 48 h after PACU discharge, and on postoperative day (POD) 30, at rest and during coughing. The AUC of NRS pain scores was calculated using the standard methods. QoR-15 scores were assessed at 18:00–20:00 on POD 1 and 2. Episodes of nausea and vomiting in the PACU and during the first two postoperative days were recorded. Nausea severity was scored on a 4-point scale (0 = none, 1 = mild, 2 = moderate, 3 = severe). Rescue antiemetics (metoclopramide 5 mg i.v.) were available if nausea score ≥ 2. HADS was scored at 18:00–20:00 on POD 1, 2 and on POD 30. Subjective postoperative sleep quality was assessed using NRS at 08:00–10:00 on POD 1–3 and using PSQI on POD 30. Sedation/agitation was assessed using the Richmond Agitation-Sedation Scale (RASS) at PACU discharge and twice daily (08:00–10:00 and 18:00–20:00) on POD 1 and 2.

The primary non-inferiority endpoint was the AUC of NRS pain scores (AUC_NRS_) during coughing within 48 h postoperatively. The primary superiority endpoint was postoperative quality of recovery, defined as the mean QoR-15 score on POD 1 and 2. Secondary endpoints included NRS pain scores at rest and during coughing at each time point; incidence of NRS pain score > 7 during coughing and NRS pain score > 3 at rest; number of successful PCIA demands; incidence and severity of PONV; gastrointestinal function recovery time (defined as time to first flatus or first defecation after surgery); PSQI and HADS on POD 30. Safety endpoints included RASS, hypotension, bradycardia and drug-related psychiatric symptoms (dizziness, hallucinations, diplopia, hypertonia and nightmares).

### Sample-size estimation

Based on our pilot study of 30 patients, the mean AUC_NRS_ during coughing over 48 h was 160 (standard deviation [SD]: 41) and mean QoR-15 was 105 (SD: 10) after gynaecological laparoscopic surgery with opioid-based PCIA. For non-inferiority testing of AUC_NRS,_ a margin of 24 was selected, corresponding to a mean difference of approximately 0.5 NRS units per hour over 48 h. A delta of 0.5 on the 0–10 NRS is considered an exacting yet clinically acceptable threshold in pain research, and a smaller difference would be imperceptible to patients and unlikely to alter analgesic management. This margin is also consistent with Wang et al. [18], who used a cumulative AUC margin of 26.5 for VAS scores in a 48-h non-inferiority trial of opioid-free analgesia after hepatectomy. With 90% power and one-sided α = 0.025, 126 patients were required. For superiority testing of QoR-15, we assumed esketamine-dexmedetomidine based opioid-free analgesia could increase the score by 6 points [19] with 90% power; 120 patients were needed. We selected the larger sample size and allowed for a 6% dropout rate, giving a target recruitment of 134 patients.

### Statistical analysis

A co-primary endpoint strategy was prespecified in the statistical analysis plan. The study was considered to have met its primary objectives only if both primary endpoints achieved statistical significance. Because this joint-testing strategy requires both endpoints to be significant simultaneously, the overall type I error rate is controlled at or below the nominal level without the need for additional multiplicity adjustment. For non-inferiority testing, AUC_NRS_ was compared using one-sided two-sample *t*-tests after normality assessment. Non-inferiority was confirmed if the upper bound of the one-sided 95% confidence interval (CI) for the between-group difference did not exceed the predefined non-inferiority margin, with a one-sided significance level of 0.025. For comparison of QoR-15 scores between groups, the unpaired *t*-test was used.

For secondary endpoints, normally distributed data were compared using unpaired *t*-tests; between-group differences were expressed as mean difference and 95% CI. Non-normally distributed data and ordinal data were compared using the Mann–Whitney *U* test; median differences and 95% CI were calculated using Hodges-Lehmann estimators. Categorical variables were analysed using *χ^2^* tests or Fisher’s exact test; relative differences were expressed as risk ratios (RR) and 95% CI. For the secondary analysis of QoR-15 domains, between-group comparisons were performed for each of the five domains, and a two-tailed P-value < 0.01 was considered statistically significant to account for multiple comparisons using the Bonferroni method. For all other secondary endpoints, no adjustment for multiplicity was applied, and these findings should be considered exploratory.

The primary analysis was based on the intention-to-treat (ITT) population, excluding only patients who withdrew consent or had cancelled surgery. A per-protocol (PP) analysis was also performed for the primary outcome after excluding patients with major protocol violations. No interim analyses were planned. Statistical analyses were performed using SPSS version 26.0 (IBM Corp., Armonk, NY, USA) and R version 4.0.2 (R Foundation for Statistical Computing, Vienna, Austria).

## Results

### Patient screen

**Figure 1.**
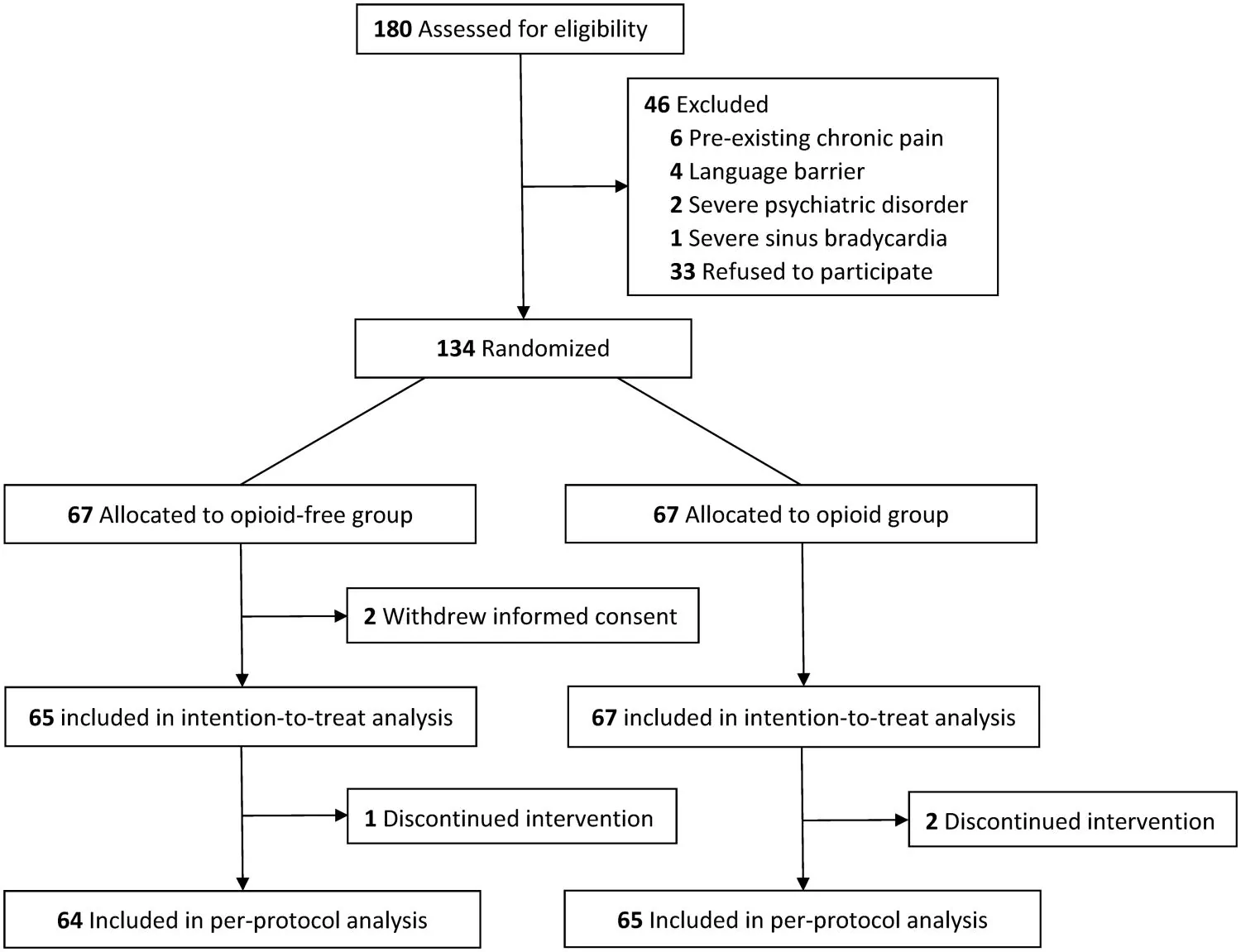
Flowchart of the trial.

Between 10 January 2024 and 15 May 2025, 180 patients were screened. Forty-six were excluded and 134 were randomized to the opioid-free or opioid group. Two patients withdrew consent before intervention and declined follow-up; three stopped PCIA within 48 h. In total, 132 patients were included in the ITT analysis and 129 in the PP analysis (Figure 1).

### Baseline characteristics and intraoperative variables

Baseline and intraoperative data are shown in Table 1. In the opioid-free group, the mean esketamine infusion rate was 12.1 (IQR 10.4–14.2) μg kg^−1^ h^−1^ and mean dexmedetomidine infusion rate was 0.036 (IQR 0.031–0.043) μg kg^−1^ h^−1^.

**Table 1.** Baseline and intraoperative data.

Variable	Opioid analgesia (*n* = 67)	Opioid-free analgesia (*n* = 65)
Demographics		
Age, mean (SD), year	39.8 (11.8)	40.5 (10.0)
BMI, mean (SD), kg m^−2^	23.5 (3.3)	24.4 (3.8)
Comorbidity, *n* (%)		
Hypertension	6 (9.0)	5 (7.7)
Diabetes mellitus	1 (1.5)	1 (1.5)
Thyroid disease	3 (4.5)	2 (3.1)
Anemia	2 (3.0)	3 (4.6)
Preoperative assessment		
ASA class, *n* (%)		
I	29 (43.3)	26 (40.0)
II	32 (47.8)	36 (55.4)
III	6 (9.0)	3 (4.6)
Pain score, median (IQR)^a^	0 (0–0)	0 (0–0)
Anxiety, median (IQR)^b^	7 (6–10)	8 (6–9)
Depression, median (IQR)^c^	5 (4–7)	5 (4–8)
Sleep quality, median (IQR)^d^	4 (2–6)	3 (2–5)
Baseline quality of recovery, mean (SD)^e^	134 (5)	135 (5)
Surgical site, *n* (%)		
Uterus only	21 (31.3)	12 (18.5)
Ovarian only	13 (19.4)	9 (13.8)
Tubal only	17 (25.4)	18 (27.7)
Uterus + Tubal	2 (3.0)	8 (12.3)
Ovarian + Tubal	3 (4.5)	3 (4.6)
Uterus + Ovarian + Tubal	11 (16.4)	13 (20.0)
Anesthetics		
Midazolam, median (IQR), mg	3 (3–3)	3 (2–4)
Propofol, median (IQR), mg	555 (400–651)	518 (354–663)
Sufentanil, median (IQR), μg	20 (15–20)	20 (15–25)
Remifentanil, median (IQR), mg	1.8 (1.3–2.1)	1.7 (1.1–2.1)
Blood loss, median (IQR), ml	20 (10–50)	20 (10–50)
Total amount of fluids, median (IQR), ml	1200 (1000–1300)	1200 (900–1500)
Duration of surgery, median (IQR), min	106 (72–135)	98 (63–141)
Duration of anesthesia, median (IQR), min	137 (96–163)	130 (97–171)

Abbreviations: ASA, American Society of Anesthesiologists; BMI, body mass index; IQR, interquartile range; QoR, quality of recovery; SD, standard deviation.

^a^Assessed by the numerical rating scale.

^b^Evaluated by the Pittsburgh Sleep Quality Index.

^c^Assessed by Hospital Anxiety and Depression Scale.

^d^Assessed by QoR-15.

### Primary outcomes

**Figure 2.**
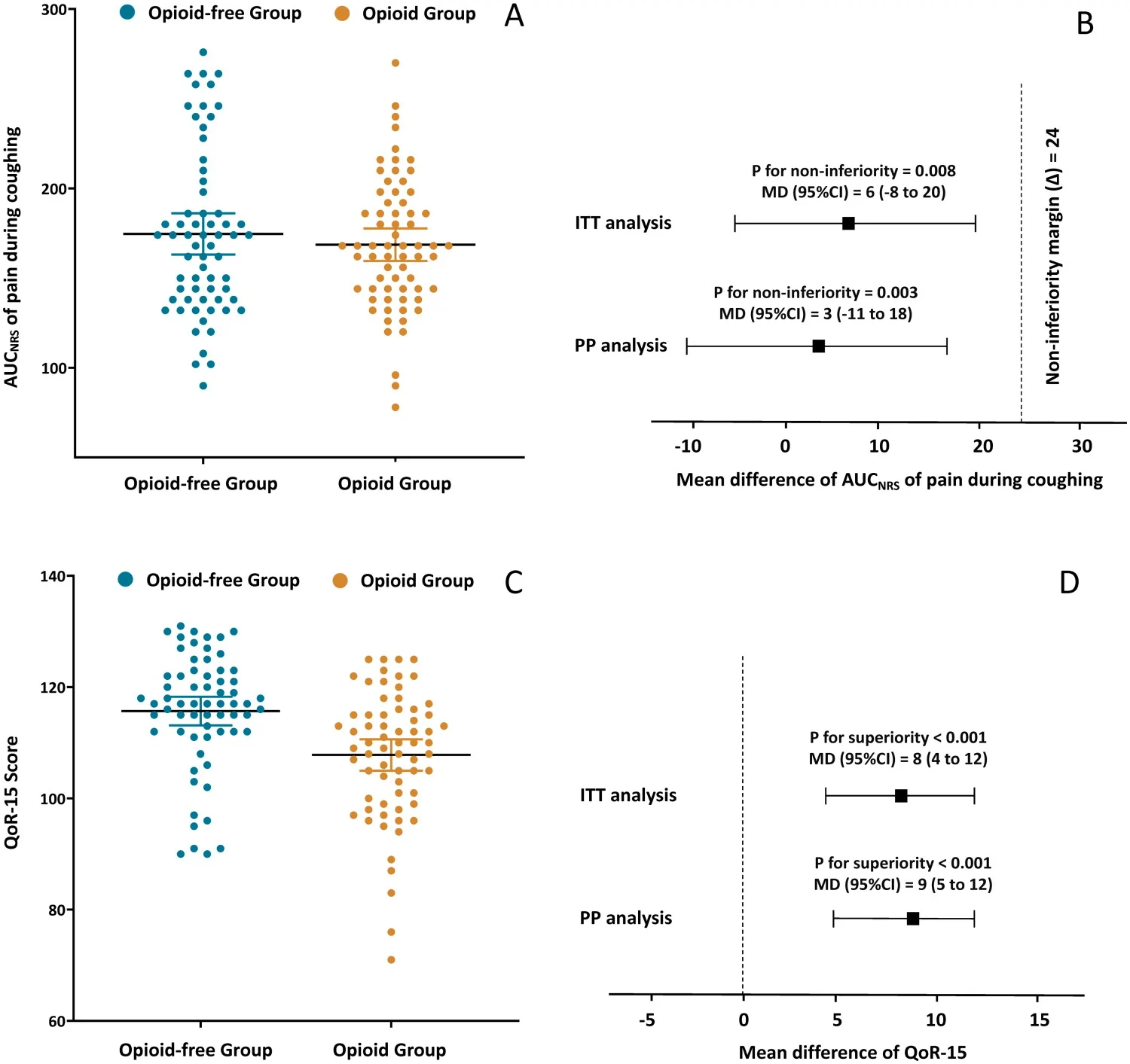
Comparison of the two primary endpoints between the two groups. (**A**) The distribution of AUC_NRS_ of pain during coughing in the two groups, where the black horizontal lines represent the means, and the green and orange horizontal lines represent the 95% CI for the means. (**B**). The non-inferiority test of AUC_NRS_ of pain during coughing between the two groups. The vertical dashed line at 24 indicates the non-inferiority margin. (**C**). The distribution of QoR-15 in the two groups, where the black horizontal lines represent the means, and the green and orange horizontal lines represent the 95% CI for the means. (**D**). The superiority test of QoR-15 between the two groups. AUC, area under the curve; CI, confidence interval; ITT, intention-to-treat; MD, mean difference; NRS, numerical rating scale; PP, per-protocol; QoR, quality of recovery.

The AUC_NRS_ of pain during coughing within postoperative 48 h in the opioid-free group was non-inferior to that in the opioid group (175 [47] vs. 169 [37]), with a mean difference of 6 [95%CI: −8 to 20] (Table 2 and Figure 2(A)); the upper CI limit was lower than the non-inferiority margin of 24 (*p* = 0.008 for non-inferiority; Figure 2(B)). PP analysis yielded similar findings (174 [47] vs. 170 [36]; mean difference 3 [95% CI −11 to 18]; *p* = 0.003 for non-inferiority; Table 2, Figure 2(B)).

**Table 2.** Efficacy outcomes.

Variable	Opioid analgesia (*n* = 67)	Opioid-free analgesia (*n* = 65)	Estimated effect (95% CI)	*P* value
**Primary outcome 1**				
AUC_NRS_ during coughing within postoperative 48 h, mean (SD)	169 (37)	175 (47)	Mean D: 6 (−8 to 20)	P for non-inferiority = 0.008
AUC_NRS_ during coughing within postoperative 48 h, mean (SD), (*per-protocol analysis*)	170 (36) *n* = 65	174 (46) *n* = 64	Mean D: 3 (−11 to 18)	P for non-inferiority = 0.003
**Primary outcome 2**				
QoR-15, mean (SD)	108 (12)	116 (10)	Mean D: 8 (4 to 12)	P for superiority <0.001
QoR-15, mean (SD) (*per-protocol analysis*)	107 (12)	116 (10)	Mean D: 9 (5 to 12)	P for superiority <0.001
**Secondary outcomes**				
Pain during coughing^a^				
Discharge from PACU, median (IQR)	3 (2–4)	3 (2–4)	Median D: 0 (0 to 1)	0.205
12 h after PACU discharge, median (IQR)	4 (3–4)	4 (3–5)	Median D: 0 (0 to 1)	0.421
24 h after PACU discharge, median (IQR)	4 (3–5)	4 (3–5)	Median D: 0 (0 to 0)	0.942
36 h after PACU discharge, median (IQR)	3 (3–4)	3 (2–4)	Median D: 0 (0 to 0)	0.818
48 h after PACU discharge, median (IQR)	3 (2–4)	3 (2–4)	Median D: 0 (0 to 0)	0.748
Incidence of VAS score >7 during coughing within 48 h after PACU discharge, n (%)	2 (3.0)	5 (7.7)	RR: 2.57 (0.54 to 12.23)	0.270
Pain at rest^a^				
Discharge from PACU, median (IQR)	2 (1–2)	2 (1–3)	Median D: 0 (0 to 1)	0.315
12 h after PACU discharge, median (IQR)	2 (2–3)	3 (2–3)	Median D: 0 (0 to 1)	0.146
24 h after PACU discharge, median (IQR)	2 (2–3)	2 (2–3)	Median D: 0 (0 to 0)	0.916
36 h after PACU discharge, median (IQR)	2 (1–3)	2 (1–3)	Median D: 0 (0 to 0)	0.879
48 h after PACU discharge, median (IQR)	1 (1–2)	1 (1–2)	Median D: 0 (0 to 0)	0.901
Incidence of NRS score >3 at rest within 48 h after PACU discharge, *n* (%)	17 (25.4)	19 (29.2)	RR: 1.14 (0.67 to 1.94)	0.697
Number of successful PCA triggers, median (IQR)	3 (1–6)	5 (1–10)	Median D: 1 (0 to 3)	0.112
The total amount of PCA within 48 h, median (IQR)	54 (50–60)	58 (50–68)	Median D: 2 (0 to 6)	0.112
Nausea				
Within 48 h after PACU discharge, *n* (%)	19 (28.4)	7 (10.8)	RR: 0.39 (0.17 to 0.89)	0.011
Nausea score, *n* (%)			Median D: 0 (0 to 0)	0.009
0	48 (71.6)	58 (89.2)		
1	2 (3.0)	2 (3.1)		
2	8 (11.9)	3 (4.6)		
3	9 (13.4)	2 (3.1)		
Vomiting				
Within 48 h after PACU discharge, *n* (%)	7 (10.4)	2 (3.1)	RR: 0.30 (0.07 to 1.30)	0.165
Anxiety^b^				
24 h after PACU discharge, median (IQR)	6 (4–9)	5 (3–7)	Median D: −1 (−2 to 0)	0.027
48 h after PACU discharge, median (IQR)	5 (3–7)	4 (3–5)	Median D: −1 (−2 to 0)	0.016
Depression^b^				
24 h after PACU discharge, median (IQR)	4 (3–5)	3 (3–5)	Median D: 0 (−1 to 0)	0.144
48 h after PACU discharge, median (IQR)	4 (3–6)	3 (3–4)	Median D: 0 (−1 to 0)	0.035
Subjective sleep quality^c^				
Surgery day, median (IQR)	5 (4–6)	6 (5–7)	Median D: 1 (0 to 1)	0.007
POD 1, median (IQR)	6 (5–7)	7 (6–8)	Median D: 1 (1 to 2)	<0.001
POD 2, median (IQR)	7 (6–8)	8 (7–9)	Median D: 1 (1 to 2)	<0.001
Gastrointestinal function recovery time^d^, median (IQR), hour	19 (16–22)	17 (14–20)	Median D: −2 (−4 to −1)	0.005
Follow-up on POD 30^e^	[3]	[2]		
Pain at rest^a^, median (IQR)	0 (0–0)	0 (0–0)	Median D: 0 (0 to 0)	0.515
Pain at coughing^a^, median (IQR)	0 (0–0)	0 (0–0)	Median D: 0 (0 to 0)	0.539
Anxiety^b^, median (IQR)	4 (3–6)	4 (3–5)	Median D: 0 (−1 to 0)	0.274
Depression^b^, median (IQR)	3 (2–5)	3 (2–4)	Median D: 0 (−1 to 0)	0.336
Sleep quality^f^, median (IQR)	4 (2–5)	4 (3–6)	Median D: 0 (−1 to 1)	0.627

Abbreviations: AUC, area under the curve; D: difference; IQR, interquartile range; NRS, numerical rating scale; PACU, post-anaesthesia care unit; PCA, patient-controlled analgesia; POD, postoperative day; QoR, quality of recovery; RR, relative risk; SD, standard deviation.

^a^Assessed by the numerical rating scale.

^b^Assessed by Hospital Anxiety and Depression Scale.

^c^Evaluated by the numerical rating scale.

^d^Defined as time to first flatus or first defecation after surgery.

^e^Numbers in square brackets indicate the number of patients with missing data.

^f^Evaluated by the Pittsburgh Sleep Quality Index.

The mean global QoR-15 score on POD 1 and 2 was significantly higher in the opioid-free group (116 [10] vs. 108 [12]; mean difference 8 [95% CI 4 to 12]; *p* < 0.001; Table 2, Figure 2(C)). PP analysis confirmed these results (116 [10] vs. 107 [12]; mean difference 9 [95% CI 5 to 12]; *p* < 0.001; Table 2, Figure 2(D)). Domain specific QoR-15 scores are presented in Supplemental Table 1. The opioid-free group had significantly higher scores for physical comfort and emotional state (both *p* < 0.01). There were no significant differences in pain, physical independence or psychological support (all *p* > 0.01).

### Secondary outcomes

For secondary endpoints, there were no significant between-group differences in NRS pain scores at rest or during coughing at any time point, or in the number of successful PCIA demands or total PCIA use within 48 h. Compared with the opioid group, the opioid-free group had lower incidence of nausea within 48 h of PACU discharge, lower HADS-Anxiety scores on POD 1 and 2, lower HADS-Depression scores on POD 2, better sleep quality on the night of surgery and on POD 1 and 2, and shorter time to gastrointestinal recovery. There were no between-group differences at the 30-day follow-up (Table 2).

### Safety outcomes

For safety endpoints, the incidence of adverse events was comparable between the two study groups. No psychiatric adverse events were observed in any patient, and no serious adverse events were documented throughout the study period (Table 3).

**Table 3.** Adverse events.

Variable, n (%)	Opioid analgesia (*n* = 67)	Opioid-free analgesia (*n* = 65)	*P* value
Hypotension^a^,	0 (0.0)	0 (0.0)	>0.999
Hypertension^b^	3 (4.5)	2 (3.1)	>0.999
Bradycardia^c^	2 (3.0)	4 (6.2)	0.437
Tachycardia^d^	2 (3.0)	1 (1.5)	>0.999
Desaturationx^e^	0 (0.0)	0 (0.0)	>0.999
Deep sedation^f^	0 (0.0)	0 (0.0)	>0.999
Respiratory depression^g^	0 (0.0)	0 (0.0)	>0.999
Dizziness^h^	6 (9.0)	7 (10.8)	0.727
Constipation^i^	1 (1.5)	1 (1.5)	>0.999
Pruritus^j^	2 (3.0)	0 (0.0)	0.496
Psychiatric symptoms	0 (0.0)	0 (0.0)	>0.999
Hallucination^k^	0 (0.0)	0 (0.0)	>0.999
Diplopia^l^	0 (0.0)	0 (0.0)	>0.999
Hypertonia^m^	0 (0.0)	0 (0.0)	>0.999
Dreaminess^n^	0 (0.0)	0 (0.0)	>0.999
Nightmare^o^	0 (0.0)	1 (1.5)	0.492

^a^Systolic blood pressure <90 mm Hg or >30% lower than baseline.

^b^Systolic blood pressure >180 mm Hg or >30% higher than baseline.

^c^Heart rate <45 beats per min.

^d^Heart rate >100 beats per min.

^e^Pulse oxygen saturation (breathing air) <90%.

^f^Ventilatory frequency <10 breaths per minute.

^g^Richmond Agitation-Sedation Scale ≤-3.

^h^Sensation of spinning, lightheadedness, or unsteadiness, lasting ≥5 min.

^i^No defecation within 72 h after surgery, or difficult/painful bowel movements.

^j^Spontaneous skin or mucosal itching (with or without scratching), no obvious rash.

^k^Perception of non-existent objects (visual, auditory, etc.), confirmed by patient report or clinical observation.

^l^Seeing double images when viewing objects.

^m^Increased muscle tone, characterized by rigid or stiff limbs that resist passive movement (excluding preoperative neuromuscular disorders).

^n^Frequent and vivid dreams reported postoperatively, affecting sleep continuity.

^o^Frightening or anxiety-provoking dreams that cause awakening.

## Discussion

In this randomized controlled trial of patients undergoing gynaecological laparoscopic surgery, postoperative opioid-free analgesia combining esketamine and dexmedetomidine was non-inferior to sufentanil-based opioid analgesia for pain control during the first 48 h postoperatively. Furthermore, postoperative quality of recovery was superior in the opioid-free group, with comparable scores in the pain domain but marked improvements in physical comfort and emotional state.

Dexmedetomidine and esketamine are commonly used as postoperative analgesic adjuncts, with distinct analgesic mechanisms. Esketamine exerts analgesia mainly by blocking central N-methyl-D-aspartate receptors [20], whereas dexmedetomidine produces analgesia by activating α_2_-adrenoceptors in the spinal dorsal horn, thereby reducing nociceptive input and transmission [21]. Zhao et al. [22] reported that adding 0.72 mg kg^−1^ esketamine to PCIA improves analgesic efficacy in elderly patients undergoing total hip or knee arthroplasty. Meta-analyses have also shown that dexmedetomidine added to postoperative PCIA significantly reduces pain scores and opioid consumption [7]. Zhang et al. [23] demonstrated that a low-dose combination of esketamine and dexmedetomidine (0.25 mg ml^−1^ esketamine and 1 μg ml^−1^ dexmedetomidine) safely improves analgesia after scoliosis correction. To our knowledge, this is the first trial to investigate dexmedetomidine–esketamine for postoperative opioid-free analgesia, and we found analgesic efficacy non-inferior to opioid-based regimens. This finding may be associated with relatively minor surgical trauma of laparoscopic surgery and routine use of NSAIDs in patients without contraindications in our study.

Numerous studies have examined the effects of dexmedetomidine and esketamine on postoperative recovery quality. Two high-quality meta-analyses of randomized controlled trials showed that perioperative dexmedetomidine or esketamine each significantly improve postoperative recovery quality [14,24]. With respect to sleep, a meta-analysis found that dexmedetomidine added to PCIA enhances sleep efficiency and improves sleep structure on the first two postoperative nights [25]. Qiu et al. [15] reported that intraoperative esketamine infusion reduced the incidence of postoperative sleep disturbance from 44.0% to 22.8% on POD 1 and from 19.8% to 7.6% on POD 3. Even a single low dose of esketamine 0.2 mg kg^−1^ during surgical abortion reduced postoperative sleep disturbance from 71.6% to 47.1% [26]. Our findings are consistent with these reports. For PONV, meta-analyses confirm that adding dexmedetomidine to PCIA significantly reduces its incidence [27]. In our study, only nausea incidence differed significantly, with no between-group difference in vomiting. This discrepancy may be due to the relatively small sample size. For anxiety and depression, PCIA containing esketamine 0.5–1.5 mg kg^−1^ reduces postoperative depression and anxiety across various surgical populations [22,28,29]. Dexmedetomidine is widely used for preoperative and postoperative anxiety prophylaxis and treatment [30,31], and a recent trial found that dexmedetomidine–esketamine combination provides better anxiolysis than dexmedetomidine alone [32]. Our results further support these observations. The mean global QoR-15 score on POD 1 and 2 was significantly higher in the opioid-free group (116 [10] vs. 108 [12]; mean difference 8 [95% CI 4 to 12]; *p* < 0.001). The observed 8-point improvement exceeds the established minimal clinically important difference of 6 points for the QoR-15 score [19], suggesting that this difference is not only statistically significant but also clinically meaningful. Several secondary outcomes, including reduced nausea, improved sleep quality and lower anxiety scores, were also consistent with the overall benefit of the opioid-free regimen. However, all secondary endpoints other than the QoR-15 domain analysis were prespecified but should be considered exploratory without multiplicity adjustment. These findings are presented as supportive signals rather than definitive evidence for individual secondary endpoints.

A low-dose ketamine infusion, typically administered at a rate of 0.1–0.5 mg kg^−1^ h^−1^, is recommended to improve postoperative analgesia [33,34]. However, even low-dose ketamine may cause psychiatric manifestations. Compared with racemic ketamine, esketamine is twice as potent and better tolerated. Bornemann-Cimenti et al. [35] showed that a minimal-dose esketamine infusion (15 μg kg^−1^ h^−1^) is comparable to conventional low-dose regimens (0.125 mg kg^−1^ h^−1^) in reducing opioid consumption and hyperalgesia, with fewer psychiatric side effects. In our study, mean esketamine infusion rate was 12.1 (IQR 10.4–14.2) μg kg^−1^ h^−1^. Meta-analyses indicate that standard dexmedetomidine doses for postoperative analgesia range from 0.02 to 0.06 μg kg^−1^ h^−1^ [27,36]. Although an increased risk of bradycardia has been reported, the rates of hypotension and excessive sedation are not increased [25]. In our study, dexmedetomidine infusion rate was 0.036 (IQR 0.031–0.043) μg kg^−1^ h^−1^, within the recommended range [27,36].

Opioid-free anaesthesia using esketamine and dexmedetomidine has gained considerable attention in recent years, with multiple studies confirming benefits including reduced PONV [37,38] and improved postoperative recovery [39,40]. However, intraoperative opioid-free techniques often require relatively high doses of dexmedetomidine and esketamine to suppress nociception, leading to frequent complications such as delayed emergence and bradycardia. Intraoperative dexmedetomidine and esketamine increase the incidence of postoperative over-sedation, prolonged awakening and orientation recovery, delayed PACU discharge, hypoxaemia, dreaming, nightmares and hallucinations [38,41–45]. Meta-analyses also identify bradycardia as a common adverse effect of opioid-free anaesthesia [5,46], with some trials terminated early due to this complication [43]. In our study, dexmedetomidine and esketamine were shifted from intraoperative use to postoperative analgesia. This approach retains their analgesic, antiemetic, anxiolytic, antidepressant and sleep promoting benefits while avoiding the complications of high intraoperative doses.

Regarding the side effect, esketamine activates the sympathetic nervous system to raise heart rate and blood pressure [47]. This is counterbalanced by the opposing cardiovascular actions of dexmedetomidine [48]. Dexmedetomidine may also reduce esketamine-related psychiatric symptoms [49]. In our study, we observed no increase in hypertension, tachycardia or psychiatric adverse events. We were particularly concerned that combined use might increase sedation, but since we used no long-acting sedatives intraoperatively, no loading doses postoperatively and no postoperative opioids, sedation levels were not significantly increased.

Several limitations of this study should be acknowledged. First, our study included a highly selected population of women undergoing minimally invasive surgery within a standardized multimodal ERAS pathway, and whether these findings can be generalized to patients undergoing more extensive surgical procedures or different perioperative pathways remains uncertain. Second, our population consisted solely of women, who are at high risk of postoperative sleep disturbance, anxiety and depression [50,51]. Our QoR-15 improvements were most marked in these domains. Whether similar benefits extend to men remains to be explored. Third, our dosing regimen was derived from the recent literature [23] and our pilot data. We recognize that this regimen is not definitive, and further investigation is needed to define optimal dosing for other surgical types. Fourth, most patients had benign disease without preoperative anxiety, depression or chronic pain, which may explain the lack of between-group differences at 30-day follow-up. Future studies should focus on high-risk populations to better detect longer-term effects on recovery. Fifth, although no neuropsychiatric adverse events were observed, the present sample size is insufficient to exclude uncommon adverse effects associated with esketamine or dexmedetomidine. Sixth, this was a single-centre trial, which may limit generalizability. Future multi-centre studies with larger and more diverse samples are needed to validate our findings. Nevertheless, to our knowledge, this is the first study to evaluate dexmedetomidine–esketamine combination as a postoperative opioid-free analgesic regimen. This novel strategy offers a promising approach to achieve effective analgesia while minimizing opioid-related risks in postoperative care.

## Conclusions

This trial shows that postoperative opioid-free analgesia using esketamine and dexmedetomidine provides analgesic efficacy equivalent to opioids in gynaecological laparoscopic surgery. Moreover, it enhances postoperative quality of recovery, especially in the domains of physical comfort and emotional state. Our regimen did not significantly increase adverse events. However, these findings are derived from a single-centre study with a highly selected population and require validation in larger, multi-centre trials involving more diverse patient groups and surgical settings.

## Supplementary Material

Supplemental Table 1.docx

## Data Availability

The dataset analyzed during the current study can be obtained from the corresponding author upon reasonable request.
