# Supplementary material for: Opioid-free analgesia with esketamine-dexmedetomidine versus opioid-based analgesia after gynaecological laparoscopic surgery: a double-blind, randomized, controlled trial
Source: Ann Med. 2026 Sep 16;58(1):2732292. doi: 10.1080/07853890.2026.2732292 (PMC13587535; doi:10.1080/07853890.2026.2732292)
Supplement: Supplementary file 1 [file IANN_A_2732292_SM2005.docx]

**Supplemental Table 1. The details of QoR-15 between two groups**

| **Variable, mean (SD)** |  | **Opioid analgesia (n = 67)** | **Opioid-free analgesia (n = 65)** | **Mean difference (95%CI)** | ***P* value** |
| --- | --- | --- | --- | --- | --- |
| **Total score** | POD 1  POD 2 | 103.5 (12.4)  111.5 (11.5) | 111.5 (11.6)  119.4 (9.9) | 8.0 (3.8 to 12.1)  7.9 (4.2 to 11.6) | <0.001  <0.001 |
| ***Physical comfort*^a^** | POD 1  POD 2 | 32.6 (5.5)  35.6 (4.8) | 36.5 (5.7)  39.0 (4.0) | 4.0 (2.0 to 5.9)  3.4 (1.9 to 4.9) | <0.001  <0.001 |
| Able to breathe easily | POD 1  POD 2 | 8.6 (1.0)  8.9 (0.8) | 8.5 (1.5)  8.8 (1.0) | -0.1 (-0.6 to 0.3)  0.0 (-0.4 to 0.3) | 0.646  0.824 |
| Been able to enjoy food | POD 1  POD 2 | 5.1 (2.1)  5.6 (1.7) | 6.1 (2.0)  6.3 (1.5) | 1.0 (0.2 to 1.7)  0.6 (0.1 to 1.2) | 0.009  0.014 |
| Feeling rested | POD 1  POD 2 | 5.7 (1.7)  6.5 (1.7) | 6.4 (1.8)  7.3 (1.8) | 0.7 (0.1 to 1.3)  0.8 (0.2 to 1.4) | 0.030  0.010 |
| Have had a good sleep | POD 1  POD 2 | 5.3 (1.5)  6.0 (1.7) | 6.5 (1.6)  7.3 (1.4) | 1.2 (0.7 to 1.7)  1.2 (0.7 to 1.8) | <0.001  <0.001 |
| Nausea or vomiting | POD 1  POD 2 | 7.7 (2.3)  8.6 (1.4) | 9.0 (1.2)  9.4 (0.6) | 1.2 (0.6 to 1.9)  0.8 (0.4 to 1.2) | <0.001  <0.001 |
| ***Physical independence*^a^** | POD 1  POD 2 | 9.4 (2.6)  11.1 (3.0) | 10.1 (2.2)  12.0 (2.6) | 0.6 (-0.2 to 1.5)  1.0 (0.0 to 1.9) | 0.128  0.052 |
| Able to look after personal toilet and hygiene unaided | POD 1  POD 2 | 5.4 (1.4)  6.4 (1.6) | 5.7 (1.4)  6.7 (1.8) | 0.3 (-0.2 to 0.8)  0.3 (-0.3 to 0.9) | 0.214  0.359 |
| Able to return to work or usual home activities | POD 1  POD 2 | 4.0 (1.8)  4.6 (2.0) | 4.3 (1.5)  5.3 (1.7) | 0.3 (-0.2 to 0.9)  0.7 (0.0 to 1.3) | 0.244  0.037 |
| ***Psychological support*^a^** | POD 1  POD 2 | 17.2 (1.9)  17.9 (1.9) | 17.9 (1.7)  18.4 (1.5) | 0.7 (0.1 to 1.3)  0.5 (-0.1 to 1.1) | 0.027  0.108 |
| Able to communicate with family or friends | POD 1  POD 2 | 8.7 (1.2)  8.9 (1.1) | 8.9 (1.1)  9.2 (1.0) | 0.1 (-0.3 to 0.5)  0.2 (-0.2 to 0.5) | 0.475  0.313 |
| Getting support from hospital doctors and nurses | POD 1  POD 2 | 8.5 (1.2)  8.9 (1.1) | 9.1 (1.1)  9.2 (1.0) | 0.6 (0.2 to 1.0)  0.3 (-0.1 to 0.6) | 0.005  0.115 |
| ***Emotional state*^a^** | POD 1  POD 2 | 28.4 (5.1)  30.2 (4.7) | 31.2 (3.9)  33.4 (3.6) | 2.8 (1.2 to 4.3)  3.2 (1.8 to 4.7) | <0.001  <0.001 |
| Feeling comfortable and in control | POD 1  POD 2 | 6.6 (1.5)  7.0 (1.6) | 7.3 (1.4)  8.0 (1.5) | 0.7 (0.2 to 1.2)  1.0 (0.4 to 1.5) | 0.005  <0.001 |
| Having a feeling of general well-being | POD 1  POD 2 | 6.6 (1.6)  7.0 (1.6) | 7.3 (1.6)  7.9 (1.5) | 0.8 (0.2 to 1.3)  0.9 (0.3 to 1.4) | 0.006  0.002 |
| Feeling worried or anxious | POD 1  POD 2 | 7.6 (1.5)  8.0 (1.4) | 8.3 (1.3)  8.8 (1.0) | 0.7 (0.2 to 1.2)  0.8 (0.2 to 1.3) | 0.005  <0.001 |
| Feeling sad or depressed | POD 1  POD 2 | 7.7 (1.5)  8.0 (1.4) | 8.3 (1.1)  8.8 (1.0) | 0.6 (0.1 to 1.0)  0.5 (0.1 to 0.9) | 0.013  0.008 |
| ***Pain*^a^** | POD 1  POD 2 | 15.9 (3.1)  16.8 (2.8) | 15.8 (3.3)  16.6 (3.3) | -0.1 (-1.2 to 1.0)  -0.2 (-1.2to 0.9) | 0.846  0.762 |
| Moderate pain | POD 1  POD 2 | 7.3 (1.8)  7.8 (1.8) | 7.2 (1.9)  7.6 (2.2) | -0.1 (-0.7 to 0.6)  -0.2 (-0.9 to 0.5) | 0.832  0.582 |
| Severe pain | POD 1  POD 2 | 8.6 (1.5)  9.0 (1.2) | 8.6 (1.6)  9.0 (1.4) | 0.0 (-0.6 to 0.5)  0.0 (-0.4 to 0.5) | 0.874  0.892 |

CI, confidence interval; POD, postoperative day; SD, standard deviation

^a^ Since the QoR-15 has 5 domains, comparisons were made for each domain separately. Per Bonferroni correction, differences were statistically significant at P < 0.01.
